# The Oxidative Response of Human Monocytes to Surface Modified Commercially Pure Titanium

**DOI:** 10.3389/fimmu.2021.618002

**Published:** 2021-06-02

**Authors:** Robert P. De Poi, Michael Kowolik, Yoshiki Oshida, Karim El Kholy

**Affiliations:** ^1^ Division of Dentistry, Medicine and Health Science, University of Melbourne, Melbourne, VIC, Australia; ^2^ Department of Periodontology, Indiana University School of Dentistry, Indianapolis, IN, United States; ^3^ Dental Materials Division, Department of Restorative Dentistry, Indiana University School of Dentistry, Indianapolis, IN, United States; ^4^ Department of Oral Medicine Infection and Immunity, Harvard University School of Dental medicine, Boston, MA, United States

**Keywords:** titanium, monocytes, surface roughness, wettability, surface energy, chemiluminescence, oxidative response, dental implants

## Abstract

Cellular responses to implanted biomaterials are key to understanding osseointegration. The aim of this investigation was to determine the *in vitro* priming and activation of the respiratory burst activity of monocytes in response to surface-modified titanium. Human peripheral blood monocytes of healthy blood donors were separated, then incubated with surface-modified grade 2 commercially pure titanium (CPT) disks with a range of known surface energies and surface roughness for 30- or 60-min. Secondary stimulation by phorbol 12-myrisate 13-acetate (PMA) following the priming phase, and luminol-enhanced-chemiluminescence (LCL) was used to monitor oxygen-dependent activity. Comparison among groups was made by incubation time using one-way ANOVA. One sample from each group for each phase of the experiment was viewed under scanning electron microscopy (SEM) and qualitative comparisons made. The results indicate that titanium is capable of priming peripheral blood monocytes following 60-min incubation. In contrast, 30 min incubation time lead to reduced LCL on secondary stimulation as compared to cells alone. At both time intervals, the disk with the lowest surface energy produced significantly less LCL compared to other samples. SEM examination revealed differences in surface morphology at different time points but not between differently surface-modified disks. These results are consistent with the hypothesis that the titanium surface characteristics influenced the monocyte activity, which may be important in regulating the healing response to these materials.

## Introduction

The use of endosseous dental implants to support restorations replacing missing teeth has become well established since the introduction of titanium dental implants (1, 2). The successful incorporation and rigid fixation of an implant within the surrounding bone was defined as osseointegration (3). While implants with surface characteristics that allow osseointegration have been available for many years, the exact surface characteristics necessary for optimal osseointegration remain to be completely elucidated, although it is known that a key feature is the highly stable passivating layer of titanium oxide that covers the titanium surface (4). It is also thought that the combined effects of surface energy, chemistry, and topography may play a major role during the initial phases of the biological response to the implant (5–7).

The effect of surface roughness, in particular, has been evaluated in multiple investigations. In *vivo* studies have demonstrated that increasing surface roughness of an implant results in an increase in bone to implant contact (8, 9). The vascular nature of bone and the inevitable surgical trauma created by the implant site preparation, ensures that the first tissue to come into contact with an endosseous implant is blood with its complement of inflammatory cells (10–12). While these early interactions between the inflammatory cells and the implant surface are thought to be important, much still needs to be determined about the nature of these interactions.

The cells of the mononuclear phagocyte system play a crucial role in the regulation of chronic inflammation and wound healing. Monocytes are also thought to have a significant role in the regulation of osseous metabolism, both in bone resorption and bone formation (13). Monocyte migration and spreading is influenced by the surface energy and roughness of the material on which the cells are attached (14, 15). It has been shown that macrophage attachment to surfaces increases with increasing surface roughness (16). More recently, it was found that the number of monocytes attached to blasted titanium surfaces was significantly greater than to machined titanium surfaces (17). These observations suggest that macrophage adherence may provide signals that induce specific macrophage functions (18). There is concern, however, that attachment of monocytes/macrophages to implant surfaces *in vivo* may jeopardize successful osseointegration since these cells are capable of inducing bone resorption and chronic inflammation.

Initially, the monocyte is primed by a low-grade stimulus, which elevates the cell to a heightened but subthreshold level of activation. Once activated, primed monocytes undergo respiratory burst activity and generate enhanced levels of reactive oxygen species and have higher levels of degranulation and greater phagocytic activity when compared to resting state monocytes. The respiratory burst, through the generation of reactive oxygen species, produces chemiluminescence (CL). The level of activity can be measured in a chemiluminometer. It is possible that the level of monocyte priming, and activation may be directly linked to the rate of healing following implant placement and long-term stability of the rigid bone-implant interface, although this has not been verified. Since the attachment of cells to a titanium surface is an important phenomenon in the area of clinical implant dentistry, a major consideration in designing implants has been to produce surfaces that promote desirable responses in the cells and tissues. Following machining, the surface roughness of an implant may be altered by mechanical methods such as mechanical polishing and sandblasting or by chemical methods, which include anodizing, etching and coating. These processes can change the surface properties of the commercially pure titanium (CPT). In particular, surface roughness and surface energy or wettability, measured as contact angle, have been shown to be affected by various surface conditioning treatments of grade 2 CPT (19). These fine features are significant in promoting osteoblast adherence, bone formation and attachment to the implant surface (20, 21).

The purpose of this *in vitro* study was to determine the priming and activation levels of the respiratory burst activity of human peripheral blood monocytes in response to surface-modified grade 2 CPT disks with a range of surface energies and surface roughness. Secondly, to envisage by scanning electron microscopy (SEM), the surface characteristics of these human peripheral blood monocytes adherent to the surface-modified CPT. It was hypothesized that human peripheral blood monocytes have a greater priming and respiratory burst activation response to CPT of higher surface energy than to those of lower surface energy; and secondly, that human peripheral blood monocytes have a greater priming and respiratory burst activation response to CPT of greater roughness than to those of lesser roughness. We postulate that the level of monocyte priming, and activation may be linked to the rate and type of healing following implant placement and the long-term stability of the rigid bone-implant interface. It is thought that a specific range of monocyte priming and activation levels may be conducive to a healing rate that allows the bone to be properly organized and mineralized allowing the development of a rigid bone-implant interface that will be stable under loading for a long period of time and further, that surface energy and surface roughness of the biomaterial will determine the level of priming and respiratory burst activity of the adherent monocytes.

## Material and Methods

### Isolation of Human Blood Monocytes

Whole blood was collected from healthy adult donors at the Central Indiana Regional Blood Center in Indianapolis (CIRBC), Indiana, and purchased under an IRB-approved contract. No distinctions were made between race, age or sex. Health is routinely determined retrospectively by testing for standard infectious diseases according to a standard CRIBC protocol. Blood (1 unit or 470 ml/experiment) was collected in citrate phosphate dextrose solution anticoagulant bags and centrifuged at 2000x g at 4°C for 4 min. Buffy coat layers were drawn off by the blood center and were used as the source of monocytes. Once in the laboratory, the buffy coat was diluted in a 1:1 ratio with RPMI (Sigma Chemicals; St. Louis, MO) to maximize efficiency of separation. Subsequently the monocytes were isolated from the buffy layer by a variation of the double dextran method (22–25). This involved placing 3 ml of HISTOPAQUE-1119 (Sigma Chemicals), a density gradient cell separation medium, in 15 ml conical test tubes at room temperature. Next, 3 ml of HISTOPAQUE-1077 (Sigma Chemicals) was carefully layered on with a pipette, and 6 ml of buffy coat/RPMI mixture was layered on top very carefully, so as to not disrupt the histopaque layers. The buffy coats and separation medium were then centrifuged at 1700 rpm for 35 min at room temperature (18-26°C). Following centrifugation, the plasma was on top of the mononuclear layer that was above a cloudy layer containing clumped cells and separating medium, which was above the granulocyte layer. The layer containing granulocytes sits directly on top of the heavy Red Blood Cell (RBC) layer. This lower layer is a pellet of RBC. Following centrifugation, the monocyte layer was drawn off with a bulb pipette and washed twice with 10 ml of Phosphate Buffered Saline (PBS), then centrifuged at 950 rpm for 10 min, and the supernatant discarded. This wash was then repeated with 10 ml RPMI. The cells were then resuspended in 10 ml RPMI. The cell suspension was placed in a polystyrene culture dish, covered and incubated at 37°C for at least 1 h. The culture dish was then gently shaken and the non-adherent cells (lymphocytes) were removed by pipetting and rinsing with warm (37°C) RPMI medium. The monocytes were then rinsed with a Calcium and Magnesium-free Hanks Balanced Salt solution (Sigma Chemicals) and detached by scrapping the cells off the petri dish with a sterile cell-lifter instrument (Fisher Scientific; Itasca IL). The cells were then pelletized by centrifugation at 950 rpm for 10 min at 4°C, and resuspended in 10 ml RPMI, stained with Trypan blue (Sigma Chemicals) and counted with a hemocytometer to determine the number of monocytes and their viability. The cells were then resuspended to a final concentration of 1.0 X 10^6^ cells/ml.

### Preparation of Specimens

Rods of CPT ASTM grade 2 were cut into 120 (5 x 5 x 1 mm) disks and prepared in similar fashion to the methods described by Lim (19). The specimens were polished using #800-grit SiC metallographic papers on all sides until a visually uniform surface was obtained. All specimens were washed in distilled water and cleaned in an ultrasonic bath and dried at room temperature. Each disk was then randomly assigned to one of six treatment groups, including the control, until there were 20 specimens in each group (**Table 1**). The particular surface modifications were chosen to reflect a range of surface roughness and wettability (**Table 2**). The six processes were divided into four treatment types: mechanical (control) to stimulate a machined surface, chemical (groups 4 and 5), mechano-chemical (group 2) and oxidation (groups 3 and 6). Following preparation, the samples were sterilized by gamma radiation (Wright Medical Technology; Arlington, TN) prior to incubation with the monocytes.

**Table 1:** List of surface modification conditions of commercially pure titanium disks.

Titanium Group	Surface modification
(1) Mechanical treatment group (control)	
1	As-polished with #800 grit SiC metallographic paper.
(2) Mechano-chemical group	
2	Sand-blasting (50 μm alumina particles) at 120 psi for 1 minute with a fixed distance (1 cm) between the sample of the surface and blasting tip, followed by chemical treatment in boiling HCl/H_2_SO_4_/H_2_O (20ml/20ml/260ml) for 3 hours.
(3) Oxidation treatment group	
3	In 70°C 5 M NaOH for 24 hours, followed by in-air oxidation at 600°C for 1 hour.
6	HF/HNO_3_/H_2_O (1/1/2 by fraction) for 10 seconds, followed by process (3).
(4) Chemical treatment group	
4	Immersed in boiling 3% H_2_O_2_ for 6 hours.
5	Boiling in 5% H_2_SO_4_ for 15 hours.

All samples (all sides) prior to the treatments were mechanically polished under the same condition as control samples (group 1).

**Table 2:** Relationship between surface roughness and contact angle on grade 2 commercially pure titanium using distilled water.

Titanium Group	R_a_ (µm)	R_max_ (µm)	Contact angle Water (°)
	(Std Dev)	(Std Dev)	(Std Dev)
1	0.57	3.41	60.19
	( ± 0.25)	( ± 2.05)	( ± 5.06)
2	1.69	9.73	58.98
	( ± 0.99)	( ± 5.85)	( ± 1.69)
3	1.52	7.52	16.88
	( ± 1.21)	( ± 6.39)	( ± 1.67)
4	0.61	3.73	58.48
	( ± 0.28)	( ± 1.19)	( ± 1.61)
5	2.15	14.14	72.99
	( ± 0.75)	( ± 5.94)	( ± 2.40)
6	2.38	14.53	10.51
	( ± 0.12)	( ± 0.93)	( ± 1.25)

*All data from Lim (Lim YJ 2000).

R_a_, Average roughness.

R_max_, Maximum roughness.

### Development of Methodology

**Figure 1:**
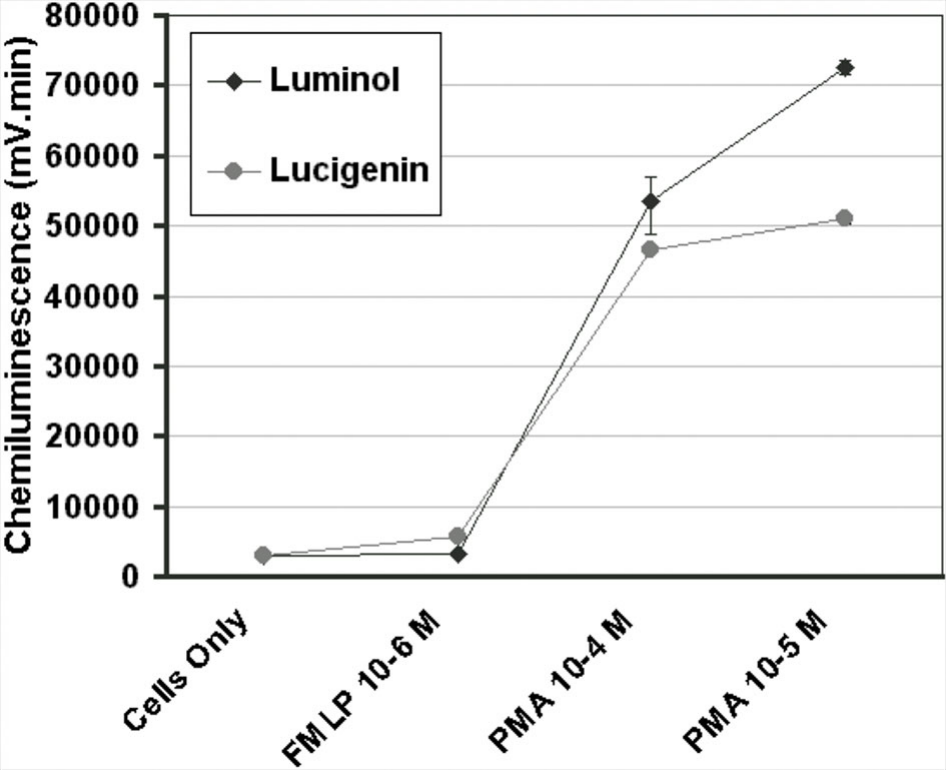
Concentration curves for PMA and FMLP in the luminescence system, as indicated by the total chemiluminescence response of monocytes activated with PMA 10^-4^ M, PMA 10^-5^ M, or FMLP 10^-6^ M with luminol 10^-6^ M or lucigenin 10^-4^ M. (n=3) (Error bars=% covariance).

After a series of pilot experiments, it was found that PMA 1 x 10^-5^ M with luminol 1 x 10^-6^ M provided the optimal conditions for a maximal cell response in our system (**Figure 1**).

### Chemiluminescence Assay 60-Minutes Incubation

The monocytes obtained from the buffy coat separation were stored at 4°C until used. 500 µl of monocyte suspension (1 x 10^6^ monocytes), 100 μl luminol 10^-6^ M and 300 µl of phosphate buffered saline (PBS) were dispensed into each reaction cuvette. One experimental or control disk was placed into each reaction cuvette with the exception of the monocyte control cuvettes and the blank control cuvette. The monocytes were incubated with the disks for 60-min at 37°C. Four repeats of each treated disk were used in each of two experimental runs giving an n of 8. There was also one cell only control in each run. The reaction was followed in a Model 1251 BioOrbit Luminometer (Turku, Finland) for 1 h representing the priming phase. After 1 h the luminometer was programmed to dispense PMA 10^-5^ M to activate the cells as a secondary stimulant. The reaction was followed for an additional 1 h representing the activation phase. Chemiluminescent output was measured in millivolts (mV) and data captured using the Multiuse Data Handling Program (BioOrbit, Turku, Finland). The integral or total mV output versus time of the monocytes was calculated and reported in mV*min.

### Chemiluminescence Assay 30-Minutes Incubation

The cuvettes containing the CPT disks were prepared in a similar manner to the first assay but in triplicates, with two runs giving an n of 6. This allowed space for a triplicate of cell only controls and one control cuvette containing only the reagents in each run. In this experiment the monocytes were incubated with the disks for 30-min at 37°C. After 30-min, the luminometer was programmed to dispense PMA 10^-5^ M to activate the cells as a secondary stimulant. The reaction was followed for an additional 90-min representing the activation phase (**Table 3**).

**Table 3:** Protocol design for chemiluminescence assay with 30-minutes priming.

Variables	Priming Phase	Activation Phase	Total CL
	Measurement Period 0-30 min ↓ 10^-6^ Luminol	Measurement Period 30-120 min ↓ 10^-5^ PMA	Measurement Period 0-120 min
Blank	1 cuvette/replicate →		
Cells Only	3 reps →		
CPT group 1	3 reps →		
CPT group 2	3 reps →		
CPT group 3	3 reps →		
CPT group 4	3 reps →		
CPT group 5	3 reps →		
CPT group 6	3 reps →		
	22 cuvettes/experiment		

### Evaluation by Scanning Electron Microscopy

One sample for each of the conditioned CPT groups with the incubated monocytes was fixed in 3% gluteraldehyde after 30-min and 60-min incubation; immediately after activation with PMA 10^-5^ M following 60-min incubation; and 60-min following activation with PMA 10^-5^ M following 30-min incubation. They were then post-fixed in osmium tetroxide, dehydrated through a graded series of ethanol, chemically dried in HMSD, attached to aluminium with epoxy resin, sputter coated with 60/40 gold/palladium alloy, and examined at 25 kV by SEM. A representative scanning electron photomicrograph was taken at a magnification of 1250x and 5000x.

### Statistical Methods

The data was summarized based on different categories of titanium disks and incubation time. Group mean and standard deviation within each of the categories was calculated. Before making the comparison between the different titanium groups, a regression model was fit to test for the significant effects of titanium group, incubation time and their potential interaction. The interaction between treatment group and incubation time was significant based on the regression model (p <0.0001), so multiple comparisons between groups by incubation time were performed. These comparisons among the groups were made using one-way ANOVA models by incubation time, with Tukey’s multiple range test, adjusted for the control level.

## Results

The monocyte LCL following 60-min incubation with the titanium samples and following 60-min activation with PMA are given in **Table 4** and for 30-min incubation and 90-min activation in **Table 5**. LCL above background levels was not detected until stimulation by PMA had occurred, indicating that the titanium surface alone did not stimulate the cells. Thus, the priming and activation phase were considered together in determining the total LCL produced. In order to adjust for the control level and reduce the change in variance between each treatment group, a proportional change from control was calculated by subtracting control value from observed value and divided by the control value. All analyses were based on these ‘adjusted’ LCL values. Further, two potential outlier samples were excluded from the analysis of the data presented.

**Table 4:** Monocyte CL for run 1 and run 2. PMA 10^-5^ M, luminol 10^-6^ M, 60-minutes priming and 60-minutes post-activation.

CPT Group	Run 1		Run 2	
	Mean monocyte CL	% Covariance	Mean monocyte CL	% Covariance
	(mV.min x 10^5^)		(mV.min x 10^5^)	
Cells Only	0.38		0.17	
1	1.58	7.93	1.09	3.78
2	1.53	2.59	1.10	4.45
3	1.45	3.60	1.05	5.46
4	1.36	2.99	0.97	14.19
5	1.18	9.71	0.82	5.34
6	1.30	1.10	1.03	3.32

**Table 5:** Monocyte CL for run 3 and run 4. PMA 10^-5^ M, luminol 10^-6^ M, 30-minutes priming and 90-minutes post-activation.

CPT Group	Run 3		Run 4	
	Mean monocyte CL	% Covariance	Mean monocyte CL	% Covariance
	(mV.min x 10^5^)		(mV.min x 10^5^)	
Blank Control	0.044		0.042	
Cells Only	2.84		0.382	1.061
1	2.43	6.53	0.326	25.874
2	2.29	3.85	0.326	1.998
3	2.34	4.68	0.322	2.234
4	2.45	3.47	0.321	5.220
5	1.51	6.21	0.229	4.770
6	2.41	0.90	0.329	1.514

**Table 6** provides a summary of the proportional change of monocyte LCL value, categorized by six different treatment groups for each of the incubation times. The patterns of the percentage change of monocyte LCL values among treatment groups were similar in each of the combined experimental runs except for the changing direction. The negative value for the 30-min incubation time indicates that the mean of the monocyte LCL was smaller than the mean of the control, while the positive value for 60-min incubation indicates that the mean of the monocyte LCL was larger than the mean of the control. The means of monocyte CL for 60-min incubation was significantly higher than zero (p-value was 0.0010, 0.0011, 0.0020, 0.0027, 0.0184 and 0.0035 for treatment groups 1, 2, 3, 4, 5, and 6, respectively), which means that the mean of LCL value in each treatment group was significantly higher than the cell only control group. However, at 30-min incubation the means of monocyte LCL values was significantly lower than zero (p <0.0001 for each group), which means the mean of LCL value in each treatment group was significantly lower than the cell only control group.

**Table 6:** Summaries of proportional change of monocyte chemiluminescence by CPT groups, under 60- or 30-minutes incubation.

CPT Group	60-minutes Incubation	30-minutes Incubation
	Mean (Std. Dev)	Mean (Std. Dev)
1	4.29 (1.30)	-0.16 (0.05)
2	4.26 (1.36)	-0.17 (0.04)
3	4.01 (1.34)	-0.17 (0.04)
4	3.70 (1.44)	-0.15 (0.04)
5	2.98 (1.00)*	-0.43 (0.05)*
6	3.76 (1.47)	-0.15 (0.01)

*significant at P <0.05.

**Figure 2:**
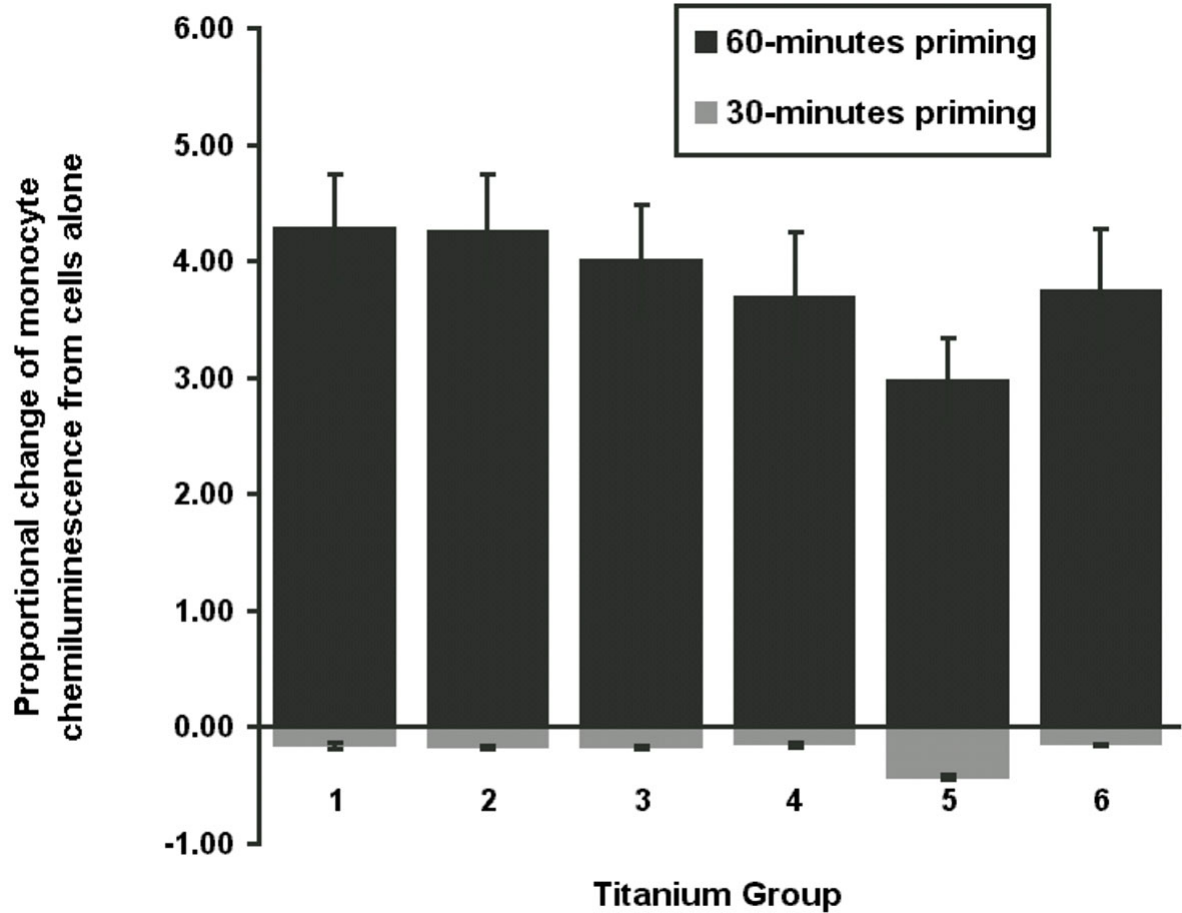
Total chemiluminescence. Figure demonstrates the proportional change of mean monocyte chemiluminescence from cells only for 30-min or 60-min priming phase. Monocyte activation was with PMA stimulation at 10^-5^ M with luminol 10^-6^ M. CPT group 5 was significantly different to the other groups (P <0.001).

The proportional change of monocyte LCL value always had the least response in group 5, regardless of experimental run or incubation time. **Figure 2** reveals that priming of monocytes incubated with group 5 at both incubation times was significantly less than the other groups (p <0.001). No significant differences between other groups were detected.

### Scanning Electron Microscopy

**Figure 3:**
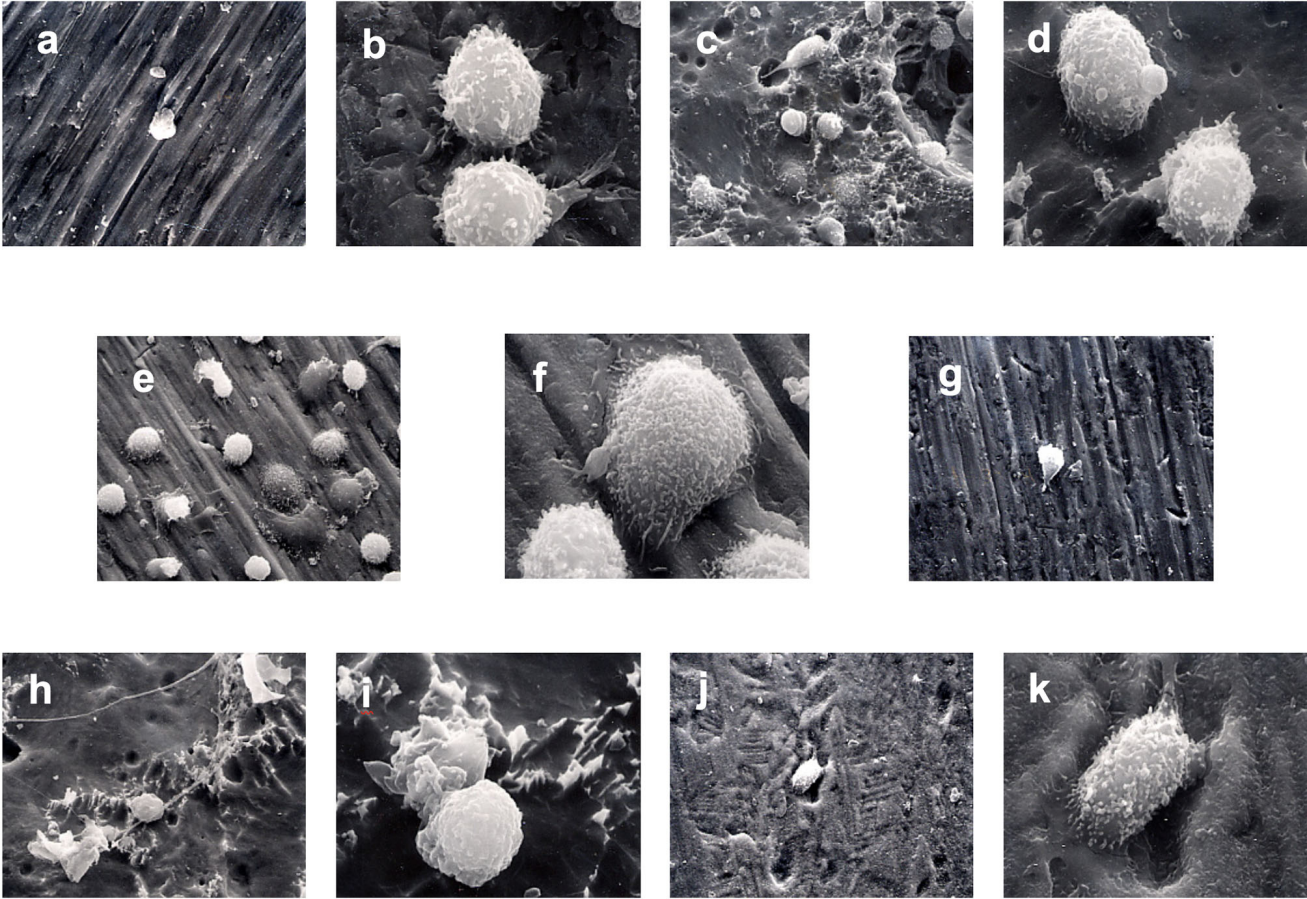
Monocyte priming. SEM photomicrographs of monocytes incubated with surface-modified titanium prior to secondary stimulation with PMA. **(A)** 1250x Group 1 30-min incubation. **(B)** 5000x Group 1 60-min incubation. **(C)** 1250x Group 2 30-min incubation. **(D)** 5000x Group 2 60-min incubation. **(E)** 1250x Group 3 30-min incubation. **(F)** 5000x Group 3 60-min incubation. **(G)** 1250x Group 4 30-min incubation. **(H)** 1250x Group 5 30-min incubation. **(I)** 5000x Group 5 60-min incubation. **(J)** 1250x Group 6 30-min incubation. **(K)** 5000x Group 6 60-min incubation.

SEM evaluation of the surface-modified CPT disks revealed differences in surface topography between treatment groups and evidence of monocyte attachment (**Figure 3**). The control disk (group 1) showed parallel scratches arising from the mechanical preparation of the titanium surface as expected. This gave the titanium surface the appearance of grooves in a regular pattern and a relatively smooth appearance. Monocytes were observed on the 30-min incubation samples, but the cells were sparsely distributed on all samples at this point. The monocytes attached to the surface in group 1 were rounded and showed some early minimal signs of membrane ruffling. The sandblasting in group 2 appeared to remove the grooved appearance created by the mechanical polishing of the control samples. Craters and pits of varying dimensions were interspersed on a surface smoothed by boiling in HCl/H_2_SO_4_/H_2_O for 3 h. Cells at various stages of attachment were observed from rounded cells, to cells with more marked membrane ruffling, to cells with pseudopodial extension, to cells that exhibited marked flattening and spreading of the cell membrane. The cells appeared to be more numerous on the areas of the surface with the most marked surface features as seen with SEM. Extension of pseudopodia outwards from the cells in several of these early time samples indicated normal function. The edges of the surface scratches were softened by the surface modification in group 3 and group 4 that produced a similar surface appearance. Pits were interspersed between an even distribution of peaks and valleys following the acid etching of group 5. For group 6, the grooved pattern was greatly reduced, and a textured surface produced by treatment with HCl/HNO_3_/H_2_O for 10 seconds followed by treatment with 70°C 5 M NaOH for 24 h, followed by in-air oxidation at 600°C for 1 h.

At 60 min incubation time and prior to activation, clumps of cells were observed attached to the group 1 surface. Extensive ruffling of the monocyte membrane and pseudopodial extension were also observed. Monocytes in group 3 exhibited a range of morphology from rounded with few pseudopodia to extensive flattening. Less ruffling of the cell membrane was noted on monocytes incubated for 60-min with group 5 than group 6 (**Figure 3**).

**Figure 4:**
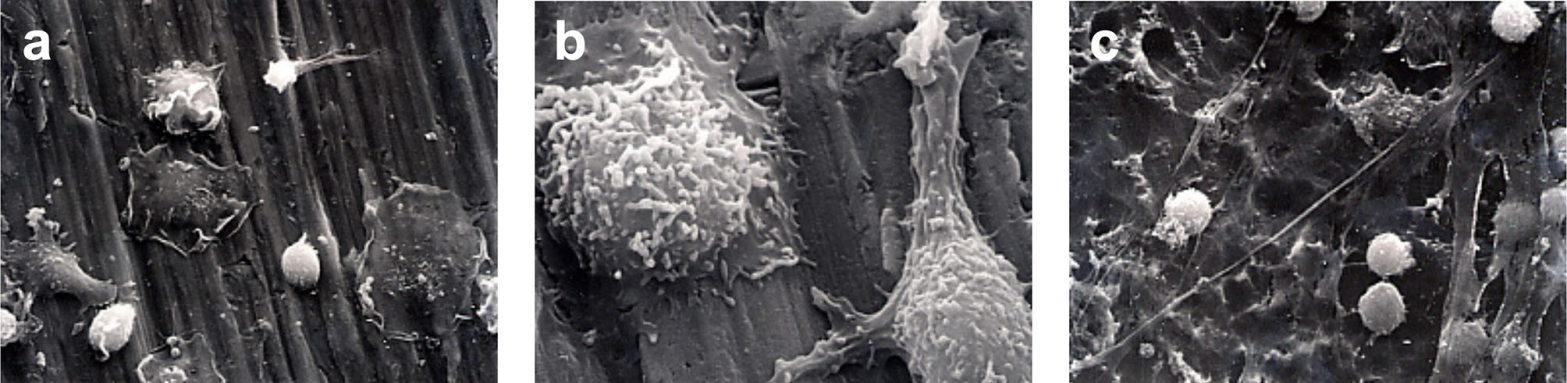
Monocyte activation. SEM photomicrographs following 60-minutes incubation and immediately following stimulation with PMA. **(A)** 1250X magnification of Group 1. Marked flattening of cells and cell membrane spreading were observed. **(B)** 5000x magnification of Group 4. Marked ruffling of the monocyte cell membrane and pseudopodial extension was demonstrated. **(C)** 1250x magnification of Group 5.

**Figure 5:**
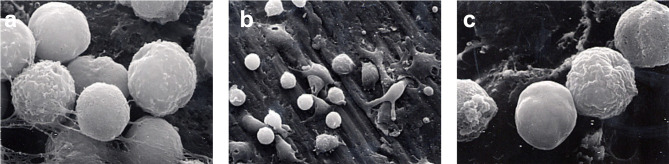
Monocyte apoptosis. SEM photomicrographs 60-min following secondary stimulation with PMA 10^-5^ M. **(A)** 5000x magnification of Group 1. The continued loss of surface features compared to earlier time periods is noted. **(B)** 1250x magnification of Group 4. Monocytes have lost the ruffled border with many cells showing signs of apoptosis. **(C)** 5000x magnification of Group 5. Loss of surface features and onset of apoptosis is evident.

Immediately following secondary stimulation with PMA, a marked flattening of cells and spreading of the cell membrane was observed for the group 1, group 4, and group 5 (**Figure 4**). A reduced level of cell membrane ruffling was observed in specimens from all other groups following PMA stimulation. At 1 h following secondary stimulation with PMA, the continued loss of surface features compared to earlier time periods was noted in all groups. This was thought to indicate the onset of apoptosis (**Figure 5**).

## Discussion

Surgical placement of any foreign biomaterial in the body elicits an acute inflammatory response, which is a vital biological response, which is regulated during early wound healing. that impacts later wound healing events such as osseointegration and guided bone regeneration (1). The impact of the early inflammatory phase on osseointegration of endosteal implants is potentially critical to wound healing and yet is still poorly understood. One of the factors possibly contributing to improved success rates of microrough implant surfaces is the reaction of the initial inflammatory cells populating the implant surface immediately after being placed in the surgically prepared osteotomy site. This primary cellular response in inflammation comes from neutrophils and monocytes. Monocytes constitute 5-10% of leukocytes and are responsible for releasing cytotoxic products that are essential for killing bacterial invaders, but these same molecules also destroy host tissues. Within These include singlet oxygen, superoxide, hydrogen peroxide and hydroxyl radicals. LCL is an exquisitely sensitive method for detecting the production of reactive oxygen species. A drawback of the method is that light emission cannot be correlated with the production of a single type of oxygen metabolite, although in monocytes LCL has been shown to be dependent on extracellular myeloperoxidase release (26). As a light emission enhancer, 100 μl of luminol at a final concentration of 1 x 10^-6^ M was used based on the preliminary concentration curve experiments that determined the optimum concentration for our system. This compares to 750 μl of luminol at a final concentration of 1 x 10^-5^ M used by McNally and Bell (27) for a similar purpose in a reaction vessel containing 1 x 10^6^ human monocytes. They also found a greater LCL response, following PMA activation, with luminol than lucigenin as was found in the present study. In our preliminary studies comparing LCL output with either PMA or FMLP as secondary stimulants, we found the highest CL output with PMA, which is similar to the findings of several other studies (28, 29).

The results of this study demonstrate that incubating human peripheral blood monocytes with surface-modified CPT results in priming or inhibition of priming in a time dependent manner. Normally, primed monocytes demonstrate higher CL output when compared to unprimed cells when subsequently challenged by a standard stimulus. The 60-min incubation lead to priming of the monocytes with the highest mean proportional change of monocyte LCL in group 1, with a mean value 4.22 times cells alone, while the lowest mean change of monocyte LCL was in group 5, with value 2.98 times cells alone. Thus, all the titanium disks had a priming effect on the monocytes at 60-minutes, increasing respiratory burst activity following stimulation by PMA as compared to cells alone. The order of the mean of percentage change of monocyte CL among CPT groups from highest to lowest was CPT1, CPT2, CPT3, CPT6, CPT4, and CPT5. This pattern was not easily interpreted in terms of surface energy and surface roughness. However, the mean percentage change of monocyte CL in group 5 was significantly lower than the other groups, indicating that the surface characteristics of group 5 lead to significantly less priming of monocytes with 60-min incubation than the other groups. CPT group 5 had the lowest surface energy of all the groups, possibly suggesting that surface energy below a certain threshold level may have an effect on monocyte priming. This finding lends support to the first hypothesis. In contrast, 30-min incubation time lead to reduced LCL on secondary stimulation as compared to cells alone for all groups. In this experiment, the highest absolute mean of proportional change of monocyte LCL was in group 5, with an absolute value of 0.43, while the lowest absolute mean of proportional change of monocyte LCL was in group 4, with an absolute value of 0.15. The order of the absolute mean change of monocyte LCL among CPT groups from highest to lowest was CPT5, CPT3, CPT2, CPT1, CPT6, and CPT4. This pattern was also not easily interpreted in terms of surface energy and surface roughness. The absolute mean change of monocyte LCL in CPT group 5 was significantly higher than the other CPT groups in both analyses. This suggests that the surface characteristics of CPT group 5 lead to a reduced CL response of monocytes with 30-min incubation time. We evaluated the relationship between surface roughness and contact angle measurement to the priming effect on human monocytes because wide differences in surface morphology of CPT are known to be the result of the various surface treatments. It may be significant that the surface modification of CPT group 5 produced the surface with the greatest contact angle; that is, the lowest surface energy of all treatments and one of the highest surface roughness levels.

Wettability on the surfaces of biomaterials has been reported to affect cell attachment considerably. It is believed that microvilli and filopodia, which work advantageously at the early stage of cell attachment, are needed for cells to pass through the energy barrier between the materials and the cells themselves (29). Many reports do not give clear definitions of wettability or adequately control other relevant factors and so make comparison difficult. As for monocytes and macrophages, it has been recognized that, in general, hydrophobic particles are better taken up by macrophages than those more hydrophilic than the phagocyte’s surface (30). Macrophages also attach more readily to hydrophobic surfaces, which contrasts with fibroblasts, which prefer hydrophilic surfaces (16). Within the range of surface roughness tested in the present investigation, no significant difference in LCL response of monocytes could be detected between rough or smooth titanium. With regards to surface roughness, CPT group 5 (high roughness) showed significant differences to CPT groups 1 and 4 (low roughness). However, CPT group 6 (high roughness) did not. The direct comparison of CPT groups with similar surface energies but different surface roughness, CPT group 2 versus CPT groups 1 and 4 showed no significant differences. Hence again while the overall pattern is not easy to interpret with regards to the effect of surface roughness on LCL response, these results would not support hypothesis 2 that human peripheral blood monocytes have a greater respiratory burst activation response to CPT of greater roughness than to those of lesser roughness. It may be that the ranges of surface roughness examined in the present investigation, from a minimum of 0.57 μm to a maximum of 2.38 μm, were not sufficiently diverse to show a difference. The range of roughness used in the present investigation was limited by the surface conditioning techniques used to prepare the samples, which may all be considered relatively smooth. Furthermore, the variation of the roughness within a particular group of CPT disks, shown by the standard deviation (**Table 2**), suggests the possibility of some overlap of roughness between groups. In fact, it has been proposed that the use of standard values, such as the mean roughness parameter R_a_, may be inadequate to describe complex topographies such as may be produced by some of the conditioning processes used in the present study (31).

SEM examination of samples at various stages of incubation time and post secondary activation revealed a wide variation in cellular response. Cells that had spread pseudopodia and had become flattened during the priming phase were observed on the surfaces of the modified titanium disks. These characteristics, which are signs of monocyte priming, were more frequently observed at 60-min than at 30-min. However, no qualitative difference in the behavior of the monocytes to different surfaces could be detected. While many studies have demonstrated that implant surface microtopography can affect cellular response for many cell types, it is also known that implant surface composition and surface energy also affect the response (32). Thus, directly correlating surface microroughness to a cellular response is a difficult task. Lincks et al. (1998) (33) determined the effect of chemical composition and surface roughness of CPT grade 2 and titanium alloy on MG63 osteoblast-like cells *in vitro*. They found that cell proliferation, differentiation, protein synthesis and local factor production were affected by surface roughness and composition. Enhanced differentiation of cells grown on rough surfaces (R_a_ 3.20-4.24 μm) compared to smooth surfaces (R_a_ 0.22-0.23 μm) was noted. Differences between CPT and titanium alloy of similar roughness were also noted indicating that factors other than roughness alone influence the cellular response.

Recently, Hayakawa et al. (2002) (32) placed CPT implants of either 1.3 μm or 14.1 μm surface roughness and with or without a calcium-phosphate coating in the femoral condyle of rabbits. They observed histologically the bone to implant contact up to 12 weeks and demonstrated similar bone to implant contact for rough or smooth CPT implants, but greater bone to implant contact was observed for the roughened and coated surfaces. This reinforces the rationale for continued research into surface treatments of implants that may alter the cellular response in a favorable manner. It is important to keep in mind that early events in a biologic response may explain effects seen much later in the healing process. Future studies concerned with the response of monocytes to roughened surfaces, may attempt to utilize surface treatments that produce a greater range of surface roughness while attempting to standardize other surface characteristics. This may assist in revealing any effect of surface roughness on monocyte CL response. Of course, it is likely that a minimum time is required before the effect of surface properties can be detected on cells and that this time is probably surface dependent and different for various cell types. Rich and Harris (1981) (16) found that it took between 1- and 7-days incubation for mouse peritoneal macrophages to show a preference to accumulate on rough surfaces, even though the macrophages were seen to move extensively over the surfaces during this time. This time requirement was shown to be an intrinsic property of the cells. Further, priming of the macrophages only slightly reduced the response time. This was in contrast to fibroblasts, which showed a preference for smooth surfaces within one day’s incubation.

Eriksson and Nygren (2001) (34) exposed CPT sheets with a water contact angle of 11° to whole blood. They found that monocytes were present on the surface in low and rather constant amounts over the 30-min to 24-h incubation time as compared to the other cell types. The monocytes covered less than 1% of the surface in this model. Furthermore, the maximal CL response was seen at 30-min incubation, which contrasts with findings of the present investigation. However, as in the present study, a CL response different to cells alone was not noted until the secondary stimulus was added.

The SEM observation of samples at the two incubation times and following activation showed cells on the surfaces of the conditioned CPT disks that had spread pseudopodia and had become flattened during the priming phase. These characteristics, which are signs of monocyte priming, were more frequently observed at 60-min than at 30-min. However, no qualitative difference in the behavior of the monocytes to different surfaces could be detected. Leake et al. (1981) (14) observed the adhesion of alveolar and peritoneal macrophages to various surfaces within 24 h. It may be that the rounded cells seen in several of the SEM in the present study were unable to locate suitable attachments sites on the CPT surface.

SEM examination also revealed higher numbers of monocytes that appeared to be undergoing apoptosis at 60-min following secondary stimulation. This may have resulted from toxic effects of the PMA or reactive oxygen species produced during the respiratory burst. Thirdly, the surface of the CPT disks themselves may have caused this effect following prolonged incubation.

Even though these experiments were of an *in vitro* and preliminary nature, they do suggest some clinical significance. The finding that incubation of human peripheral blood monocytes with variously treated CPT surfaces can induce a time-dependent variation in the LCL response and that this response may be surface dependent, suggests that the effect of CPT surfaces on monocyte response will need to be considered in the development of dental implant surfaces. It is well documented that dental implants with rough surfaces tend to show higher bone to implant contact and greater predictability where bone quality is poor (Cochran 1999) (35). It has also been shown that rough and/or hydrophilic surfaces lead to greater macrophage induced bone resorption (15). These results seem consistent with the present investigation, which has shown that CPT surfaces can prime monocytes but that the oxidative burst of these monocytes following secondary stimulation was least on the most hydrophobic surface. This leads to the possibility that there is likely to be a range of surface roughness and surface energy values that cause an appropriate level of monocyte activation, consistent with a cellular response that results in osseointegration (36). It can be speculated that the activation of monocytes outside this range is likely to result in chronic inflammation and the formation of a fibrous capsule around the implant.

It is important to note that individual variations between blood donors could significantly impact the monocyte response. The blood samples were tested for infectious diseases but not for other chronic conditions. This could be a limitation to the current investigation, however the authors tried to control for individual variation by standardizing the use of each blood sample for each experimental run involving all of the groups. Another potential limitation of the study is the lack of characterization of the purification or polarization level of monocytes. This could have helped further clarified a source of variation of cellular reactions.

Several conclusions can be made from the present study. The data clearly show that CPT is capable of priming peripheral blood monocytes following 60-min incubation. This priming of human monocytes is time dependent, with 30-min incubation leading to an inhibition of the oxidative response compared to cells alone. Secondly, the priming effect is not dependent of surface roughness within the range of roughness and incubation periods tested in this study. This suggests that CPT surfaces with roughness between 0.57 μm and 2.38 μm can prime human monocytes following 60-min incubation. Thirdly, CPT group 5 with a water contact angle of 72.99 degrees, lead to significantly reduced priming of human monocytes compared to the other groups tested. This suggests that the priming of human peripheral blood monocytes by CPT may be dependent on a critical threshold surface energy level.

## Data Availability Statement

The original contributions presented in the study are included in the article/supplementary material. Further inquiries can be directed to the corresponding author.

## Ethics Statement

The studies involving human participants were reviewed and approved by Indiana University Medical School Institutional Review Board. Written informed consent for participation was not required for this study in accordance with the national legislation and the institutional requirements.

## Author Contributions

RD and MK contributed to the development, conceptualization and execution of the project. YO contributed to development of the materials and methods and execution of the project. KE contributed to data analysis and manuscript. All authors contributed to the article and approved the submitted version.

## Conflict of Interest

The authors declare that the research was conducted in the absence of any commercial or financial relationships that could be construed as a potential conflict of interest.
